# Cognitive ability and personality as predictors of participation in a national colorectal cancer screening programme: the English Longitudinal Study of Ageing

**DOI:** 10.1136/jech-2014-204888

**Published:** 2015-02-03

**Authors:** Catharine R Gale, Ian J Deary, Jane Wardle, Paola Zaninotto, G David Batty

**Affiliations:** 1 Department of Psychology, Centre for Cognitive Ageing & Cognitive Epidemiology, University of Edinburgh, Edinburgh, UK; 2 MRC Lifecourse Epidemiology Unit, University of Southampton, Southampton, UK; 3 Department of Epidemiology and Public Health, University College London, London, UK; 4 Alzheimer Scotland Dementia Research Centre, University of Edinburgh, Edinburgh, UK

**Keywords:** COGNITION, PSYCHOLOGY, SCREENING, SOCIAL CLASS, SOCIO-ECONOMIC

## Abstract

**Background:**

The English NHS Bowel Cancer Screening Programme has offered biennial faecal occult blood testing to people aged 60–69 years since 2006, and to those aged 60–74 years since 2010. Analysis of the first 2.6 million screening invitations found that 54% of eligible people took up the invitation. The reasons for this low uptake are unclear. We investigated whether participation in screening varies according to cognitive ability and personality.

**Methods:**

Participants were members of The English Longitudinal Study of Ageing. In 2010–2011, respondents were asked about participation in bowel cancer screening, and cognitive ability and the ‘Big Five’ personality traits were assessed. Logistic regression was used to examine the cross-sectional relationships between cognitive ability and personality and screening participation in 2681 people aged 60–75 years who were eligible to have been invited to take part in the UK national screening programme for bowel cancer.

**Results:**

In age-adjusted and sex-adjusted analyses, better cognition and higher conscientiousness were associated with increased participation in cancer screening. ORs (95% CIs) per SD increase were 1.10 (1.03 to 1.18) for cognitive ability and 1.10 (1.01 to 1.19) for conscientiousness. After further adjustment for household wealth and health literacy—shown previously to be associated with participation—these associations were attenuated (ORs were 1.07 (1.00 to 1.15) and 1.07 (0.97 to 1.18), respectively).

**Conclusions:**

We found some indication that better cognitive function and greater conscientiousness may be linked with a slightly increased likelihood of participation in bowel cancer screening. These relationships need investigation in other cohorts of older people.

## Introduction

The UK National Health Service Bowel Cancer Screening Programme (BCSP) was established to improve outcomes from colorectal cancer, the third most common cause of cancer death in England.^1^ Since BCSP's initiation in England in 2006, biennial faecal occult blood testing (FOBT)—shown to reduce mortality in trials^2^—has been offered to all adults aged 60–69 years (60–74 years from 2010) registered with general practices. An analysis of participation in screening based on the first 2.6 million invitations found overall uptake was 54%.^3^ The reasons for this low uptake are unclear. Understanding which factors influence the decision to participate in bowel cancer screening is essential for designing interventions to improve the uptake of screening.

One factor which might influence whether individuals take part in bowel cancer screening is cognitive ability. People with poorer cognitive skills may have less knowledge about bowel cancer and may be less likely to comprehend the information they are given about screening and how to complete the FOBT. Two studies of older people—one based in the USA^4^ and one in Hong Kong^5^—found that those with cognitive impairment were less likely to have undergone bowel cancer screening. Although these findings provide some support for the hypothesis that cognitive function might influence participation in bowel cancer screening, it is also possible—at least in the case of the US survey which was carried out at a time when older people's access to bowel cancer screening was dependent on private insurance—that those with cognitive impairment were less likely to be recommended by their doctors to have screening.^4^ Examination of the relationship between cognitive function and participation in a national BCSP would provide a clearer indication of the relationship between cognitive ability and the decision to have screening.

There is some evidence that health literacy may determine whether individuals participate in bowel cancer screening. Health literacy has been defined as ‘the degree to which individuals have the capacity to obtain, process and understand basic health information and services needed to make appropriate health decisions’.^6^ Results of qualitative studies suggest that lack of understanding of the concept of screening or of the benefits of early detection may deter people with low literacy from undergoing bowel cancer screening.^7^ Recent findings from the English Longitudinal Study of Ageing (ELSA) indicate that people who score less than the maximum score for health literacy may be less likely to take part in the national screening programme.^8^ The relationship between health literacy and various health outcomes is at least partially explained by cognitive ability.^9–11^ Whether cognitive ability helps explain the link between health literacy and the decision to take part in bowel cancer screening is not known.

Another psychological factor that may influence the decision to have screening is personality—a largely stable set of traits and characteristics that influences thoughts, feelings and behaviour. The five-factor model of personality, consisting of the personality domains of neuroticism, extraversion, openness,^12^ agreeableness and conscientiousness, has been widely used to study the relationship between personality and health outcomes, such as mortality and health behaviours. Greater conscientiousness, for example, has consistently been linked with lower mortality and generally healthier behaviour.^13^ ^14^ There is some evidence that women who are higher in conscientiousness or openness perceive fewer barriers to undergoing cervical cancer screening.^15^ Higher conscientiousness has also been associated with having regular mammograms in a US cohort.^16^ In a study in Japan, in which respondents were assessed for neuroticism and extraversion only, the likelihood of regular participation in the National Gastric Cancer Screening Programme rose with increasing levels of extraversion and decreasing levels of neuroticism.^17^ To the best of our knowledge, there has been no investigation of the links between personality and participation in bowel cancer screening.

The ELSA is a large population-based sample of older men and women. We used these data to investigate whether participation in the national BCSP varies according to cognitive ability and personality. In addition, we examined whether cognitive ability helps to explain the link between health literacy and screening.

## Methods

### Participants

The data for this study come from the ELSA. The sample for ELSA was based on people aged ≥50 years who had participated in the Health Survey for England in 1998, 1999 or 2001.^18^ It was drawn by postcode sector, stratified by the health authority and proportion of households in non-manual socioeconomic groups. In total, 11 391 people participated in wave 1 in 2002–2003. Refreshment samples drawn from the Health Survey for England were added at waves 3 and 4 to maintain the representation of people aged 50–75 years. At wave 5 in 2010–2011, the focus of the present analyses, 9090 people took part. Ethical approval was obtained from the London Multicentre Research and Ethics Committee. Participants gave written informed consent. The research conformed to the principles embodied in the Declaration of Helsinki.

### Measures

In the wave 5 questionnaire, participants were asked whether they had ever completed a home testing kit for screening bowel cancer and whether the test was part of the NHS BCSP. These questions were added to the survey questionnaire part way through the field work. Of the 5406 participants in the wave 5 survey who were asked these questions about use of a home bowel cancer testing kit, 5399 (99.8%) provided information. Of those who had completed the testing with such a kit, <5% reported that it was not part of the BCSP. For this analysis we assumed that all those who had used the testing kit had performed so under the screening programme.

Participants took the following tests of cognitive function. Verbal memory was assessed using a test of word list learning, in which 10 common words are presented aurally on a computer and the participant is asked to recall them immediately; the word lists used for this test were used in the US Health and Retirement Study.^19^ Prospective memory was assessed using a task that required participants to remember to do a specific action at the appropriate time. Participants were told that when they were later handed a clipboard, they would need to remember to write their initials in the top left-hand corner of the page attached to the clipboard; when the appropriate point in the session was reached for the participants to carry out the action, the interviewer waited for 5 s to see if the respondent performed the correct action without a prompt. If they failed to carry out the action, the interviewer reminded them that they were going to do something and recorded what the participants then did. A correct response requires the person to carry out the correct action without being reminded. This test was previously used in the MRC Cognitive Function and Ageing Study.^20^ Attention and speed of processing was assessed using a letter cancellation task, also used in the MRC Cognitive Function and Ageing Study.^20^ The participant is given a page of random letters of the alphabet set out in rows and columns, and is asked to cross out as many target letters (P and W) as possible within 1 min. The total number of letters searched provides a measure of speed of processing. Executive function was assessed using a test of verbal (semantic) fluency taken from the CAMCOG-R.^21^ This test requires the participant to name as many animals as possible in 60 s. These cognitive tests were chosen because: they assessed cognitive processes relevant to everyday functioning; they were based on tasks sensitive to age-related cognitive decline; they avoided floor and ceiling effects; and they had been used in other studies of older people. A standardised overall measure of general cognitive ability was generated by applying principal components analysis to the test scores, extracting the first unrotated principal component that reflects the variance shared among the tests taken, and using this to calculate a score for each person. A general cognitive ability factor typically accounts for around 50% of the variance when a diverse battery of cognitive tests are given to a healthy population sample.^22^ In these data, this component accounted for 46% of the variance. Loadings of the tests on the factor were: 0.76 (verbal memory), 0.76 (verbal fluency), 0.54 (prospective memory) and 0.65 (attention).

Levels of the Big Five personality traits—extraversion, agreeableness, conscientiousness, neuroticism and openness to experience—were assessed using a version of the Midlife Development Inventory previously used in the US Health and Retirement Survey.^23^ These dimensions were measured using self-ratings of 26 adjectives. Respondents indicated the degree to which such items as ‘outgoing’, ‘caring’, ‘organised’, ‘moody’ and ‘curious’ described them, rating each one on a four-point Likert scale (ranging from 1 to 4). Each score was calculated by obtaining the average of the ratings defining that dimension. Scores were only calculated for participants who had completed more than half the items for each dimension. Cronbach α values for these data were 0.76 (extraversion), 0.80 (agreeableness), 0.68 (neuroticism), 0.67 (conscientiousness) and 0.79 (openness to experience), indicating at least adequate internal consistency.

Socioeconomic status was indexed by total net household wealth, including savings and investments, value of any property or business assets, net of debt, and excluding pension assets. Household wealth has been identified as the most accurate indicator of long-term socioeconomic circumstances in ELSA.^24^ For the current analysis, we used quintiles of total net household wealth. The mean (minimum, maximum) wealth in each of the quintiles in our analytical sample was as follows: (1) £32 119 (£93 000, £113 600); (2) £166 490 (£114 000, £207 000); (3) £255 138 (£207 006, £315 000); (4) £398 287 (£316 000, £497 200) and (5) £964 072 (£497 320, £6 556 100).

Functional health literacy was assessed using a four-item comprehension test based on instructions similar to those found on a packet of aspirin bought over a shop counter. Participants were asked to read an enlarged, fictitious medicine label. They were able to refer to the label while responding to the questions asked by the interviewer, such as “What is the maximum number of days you may take this medicine?” and “List one condition for which you might take the tablet.” The test was originally used in the International Adult Literacy Survey.^25^

### Statistical analysis

Analysis of variance and χ^2^ test were used to examine differences in characteristics according to the participation in bowel cancer screening. Spearman correlation coefficients were used to examine correlations between these characteristics. Preliminary logistic regression analyses showed that the relationship between cognitive ability or personality traits and participation in bowel cancer screening did not differ significantly by sex, so for subsequent analyses, data for men and women were pooled and adjusted for age and sex, and then for household wealth and health literacy. (A previous report on these data showed that educational attainment was not associated with participation in screening independently of household wealth.^8^ Therefore, educational attainment was not included as a covariate here.) In the logistic regression analyses we treated health literacy score as a binary variable, comparing those who gained the maximum mark to those who gained less than the maximum mark, as was done previously.^8^ Regression analyses were weighted using probability weights supplied with the data to correct for any systematic differences in response rates across subgroups.^18^ Of the 5399 people with data on whether they had participated in bowel cancer screening, 3087 were aged 60–75 years at the time of the survey and hence were within the age range who would have been invited for screening. Of these, 2681 (82%) had complete data on cognition, personality and the covariates to form our analytical sample.

## Results

Table 1 shows the characteristics of the 2681 men and women in our study according to participation in screening. Fifty-seven per cent had participated in screening. Non-participants were older, more likely to be male, had lower scores for cognitive function, lower scores for health literacy, lower levels of the personality traits openness to experience, extraversion, and conscientiousness, and were more likely to be in the lowest fifth of the distribution as regards household wealth.

**Table 1:** Characteristics of the study sample according to participation in bowel cancer screening (n=2681)

Characteristic	Participation in bowel cancer screening		
	Yes (n=1539)	No (n=1142)	p Value for difference
Age (years), mean (SD)	65.8 (3.92)	67.7 (5.08)	<0.0001
Female, n (%)	883 (57.4)	594 (52.0)	0.006
Cognitive function, mean (SD)	0.14 (1.26)	−0.19 (1.29)	<0.0001
Health literacy score, n (%)			0.002
0–3	329 (21.4)	310 (27.2)	0.001
4	1210 (78.6)	832 (72.9)	
Neuroticism, mean (SD)	2.10 (0.57)	2.10 (0.59)	0.920
Openness, mean (SD)	2.92 (0.54)	2.87 (0.55)	0.028
Extraversion, mean (SD)	3.21 (0.52)	3.15 (0.54)	0.020
Agreeableness, mean (SD)	3.53 (0.47)	3.50 (0.48)	0.172
Conscientiousness, mean (SD)	3.34 (0.46)	3.27 (0.49)	0.0022
Household wealth quintiles, n (%)			<0.0001
1 (poorest)	241 (15.7)	295 (25.8)	
2	324 (21.1)	211 (18.5)	
3	324 (21.1)	213 (18.6)	
4	324 (21.2)	210 (18.4)	
5 (richest)	325 (21.1)	213 (18.7)	

Table 2 shows the rank-order correlations between cognitive ability, health literacy, household wealth and the personality traits. Cognitive ability was moderately positively correlated with health literacy and with household wealth. It was also positively correlated, though to a lesser extent, with conscientiousness, openness and extraversion, and inversely correlated with neuroticism. Greater household wealth was also positively correlated with conscientiousness, openness and extraversion, and inversely correlated with neuroticism.

**Table 2:** Correlations† among cognitive ability, health literacy, personality traits and household wealth

	1	2	3	4	5	6	7	8
1. Cognitive ability	–							
2. Health literacy	0.29***	–						
3. Conscientiousness	0.13***	0.04*	–					
4. Extraversion	0.09**	−0.01	0.45***	–				
5. Openness	0.16***	0.04*	0.43***	0.59***	–			
6. Neuroticism	−0.06**	−0.03	−0.20***	−0.18***	−0.15***	–		
7. Agreeableness	0.01	−0.04*	0.42***	0.54***	0.40***	−0.06**	–	
8. Household wealth	0.24***	0.14***	0.11***	0.06**	0.11***	−0.08**	0.11***	–

†Spearman rank-order correlations.

*p<0.05, **p<0.01, ***p<0.001.

Table 3 shows ORs for participation in screening according to cognitive ability, personality traits, household wealth and health literacy, adjusted for age and sex. After adjustment for age and sex only, participation in screening was associated with better cognitive function and greater conscientiousness; there were no associations between the other personality traits and likelihood of participation in screening. We excluded these personality traits from further analysis as retaining them had very little effect on the associations between cognitive ability or conscientiousness and participation in screening. In a model that included cognitive ability, conscientiousness, household wealth, health literacy as well as age and sex, the association between cognitive function and participation in screening became of borderline significance (p=0.062), while that between conscientiousness and participation in screening was attenuated to non-significance (p=0.190).

**Table 3:** ORs (95% CIs) for participating in bowel cancer screening

	ORs (95% CI), separately adjusted for age and sex	ORs (95% CI), further adjusted for other covariates in the table
Cognitive function, per SD	1.10 (1.03 to 1.18)**	1.07 (1.01 to 1.15)*
Neuroticism, per SD	0.97 (0.90 to 1.05)	–
Extraversion, per SD	1.05 (0.97 to 1.13)	–
Agreeableness, per SD	1.03 (0.95 to 1.11)	–
Openness, per SD	1.04 (0.96 to 1.13)	–
Conscientiousness, per SD	1.10 (1.01 to 1.19)*	1.06 (0.98 to 1.18)
Health literacy score		
0–3	Reference	Reference
4	1.20 (0.99 to 1.74)	1.10 (0.91 to 1.34)
Household wealth, quintile		
1 (poorest)	0.56 (0.44 to 0.72)***	0.61 (0.47 to 0.79)***
2	1.10 (0.86 to 1.42)	1.16 (0.89 to 1.50)
3	1.03 (0.80 to 1.33)	1.07 (0.83 to 1.37)
4	1.02 (0.80 to 1.31)	1.04 (0.81 to 1.34)
5 (richest)	Reference	Reference

*p<0.05, **p<0.01, ***p<0.001.

We hypothesised that cognitive function might at least partially explain the previously published association in these data^8^ between health literacy and participation in bowel cancer screening. The age-adjusted and sex-adjusted OR (95% CI) for participation among those who scored the maximum mark of 4 on the health literacy measure compared to those who scored less than 4 was 1.20 (0.99 to 1.44). Further adjustment for cognitive function attenuated this estimate by 40% (OR=1.12, 95% CI 0.92 to 1.36, p=0.246).

Questions on bowel cancer screening were added to the survey questionnaire part way through data collection. As a result, 39% of participants, who had data on cognitive function, personality and the covariates, and were in the age range who would have been eligible to have been invited for screening were missing information on whether they had participated in the screening. We investigated whether participants who were not asked the questions on bowel cancer screening differed from those who were asked the questions. Those who were not asked the questions differed from those who were in having slightly lower scores for cognitive ability (mean (SD) total score for all cognitive tests combined 54.9 (10.9) vs 55.9 (11.2), p=0.003). However, there were no significant differences between these two groups in mean scores for openness (2.89 (0.55) vs 2.89 (0.54), p=0.55), neuroticism (2.07 (0.59) vs 2.10 (0.58), p=0.11), extraversion (3.16 (0.56) vs 3.18 (0.54), p=0.20), conscientiousness (3.29 (0.49) vs 3.31 (0.48), p=0.10) or agreeableness (3.51 (0.48) vs 3.52 (0.48), p=0.69), and no differences between them in the proportion who scored less than full marks on the health literacy measure (24.0 vs 23.7, p=0.830) or who were in the lowest category of household wealth (12.0 vs 10.6, p=0.08), though the latter difference was of borderline significance.

## Discussion

In this survey of men and women from the English Longitudinal Study of Ageing, those with better cognition were slightly more likely to have taken part in the National Health Service national BCSP. The effect size was small and it became of borderline significance after adjustment for household wealth and health literacy. Although higher conscientiousness was associated with a slightly greater likelihood of participation in age-adjusted and sex-adjusted analyses, this relation was attenuated after further adjustment for these covariates. Several previous studies have shown that cognitive ability explains at least part of the relationship between health literacy and health outcomes,^9–11^ and that cognitive ability and conscientiousness have been linked with future income;^26–28^ so control for household wealth and health literacy may overadjust the associations between cognitive ability and conscientiousness, and uptake of bowel cancer screening.

In a previous study using these data, Kobayashi *et al*^8^ reported that participation in bowel cancer screening was lower in men and women who gained less than full marks on a measure of functional health literacy. We found that the association between health literacy and participation in screening was attenuated by 40%, and was not statistically significant after adjustment for cognitive ability. Score on the health literacy measure was moderately correlated with cognitive ability (r=0.29). In two previous surveys where health literacy was assessed using three different measures, scores were moderately or strongly correlated with general cognitive ability, whether assessed concurrently^9^ ^11^ or in childhood.^9^ In both surveys, lower health literacy was associated with poorer health and these associations were substantially explained by cognitive ability, either alone^10^ or in combination with educational attainment and socioeconomic status.^9^ In a recent study where participants completed measures of health literacy, a battery of cognitive ability tests, and responded to questions on 12 health behaviours and health outcomes, results from an empirical factor analysis, a comparative content analysis and an incremental validity analysis gave little support to the notion that health literacy is a unique construct, leading the authors to conclude that health literacy measures are ‘simply domain-specific, contextualised measures of basic cognitive abilities’.^29^

To the best of our knowledge, no previous study has examined the link between conscientiousness and participation in a cancer screening programme. As being organised, persistent and thorough are key attributes of this personality trait, it seems plausible that individuals higher in conscientiousness would be more likely to complete the series of tasks that make up FOBT. We found that higher conscientiousness was linked with a small increase in the likelihood of having taken part in the BCSP in age-adjusted and sex-adjusted analyses—a 10% increase per SD increase.

Although a previous study of uptake of bowel cancer screening in England found evidence of a socioeconomic gradient in participation,^3^ in the current analysis the likelihood of non-participation was significantly reduced only among those people who were in the poorest quintile of net total household wealth. In contrast to the previous study where the measure of socioeconomic status was area based,^3^ we were able to use an individual-level measure which encapsulated all aspects of wealth so that our results may provide a more accurate estimate of the link between socioeconomic status and screening update.

There is considerable evidence that cognitive function tends to be poorer in those with more deprived socioeconomic backgrounds^30^ ^31^ and some evidence—rather less consistent—that personality traits vary by socioeconomic position.^31^ Several studies have found that cognitive ability and personality partially explain links between socioeconomic position and mortality.^32^ ^33^ In this study too, we found that cognitive ability and personality varied significantly by socioeconomic position—as indicated by household wealth—but adjustment for these factors had only a slight attenuating effect on the association between household wealth and participation in bowel cancer screening, suggesting that, in these data at least, they explain little of the relationship.

The strengths of our study lie primarily in its size and the fact that our sample is drawn from a cohort designed to be representative of the English population aged 50 years and over.^18^ It is the first large-scale investigation of the potential influence that cognitive ability and personality might play in the decision to participate in a BCSP. The study also has some weaknesses. Questions on bowel cancer screening were added to the survey questionnaire part way through data collection. As a result, 39% of participants who had data on cognitive function, personality and the covariates, and were in the age range who would have been eligible for screening were missing information on participation in screening. Participants who were not asked the questions on bowel cancer screening differed from those who were in having slightly lower scores for cognitive ability. There were no differences between these two groups in personality, household wealth or health literacy score. Statistical weights supplied with the data were used to minimise bias from differential non-response, but it is possible that the associations found between cognitive ability and likelihood of participating in screening may underestimate the true strength of these relationship. The cognitive tests used on the sample are few, brief, and do not include any tests of complex reasoning, which are typically those with the best loadings on general cognitive ability. This is likely to have underestimated cognition's association with screening.

In this survey of older men and women from the ELSA there was some indication that those with better cognitive ability might be more likely to participate in a national BCSP. The effect size was small and of borderline significance after adjustment for all covariates. An association between greater conscientiousness and increased likelihood of participation in screening was attenuated after full adjustment. Further investigation of the relationship between cognitive ability and personality, and uptake of bowel cancer screening in ELSA or other cohorts of older people is needed before we can properly gauge the extent to which these traits might influence participation in this screening programme.

**What is already known on this subject**

- A previous study based on data from England found that only 54% of older people who are offered faecal occult blood testing as part of the National Bowel Cancer Screening Programme take up the invitation.
- The reasons for the low uptake of screening are not known.

**What this study adds**

- Better cognition and greater conscientiousness were associated with a slightly higher likelihood of participation in the National Bowel Cancer Screening Programme, but the size of the effect was small.
